# Air particulate concentration during orthodontic procedures: a pilot study

**DOI:** 10.1186/s12903-021-01725-7

**Published:** 2021-07-21

**Authors:** Inmaculada Martín-Quintero, Alberto Cervera-Sabater, Víctor Tapias-Perero, Iván Nieto-Sánchez, Javier de la Cruz-Pérez

**Affiliations:** 1 Department of Orthodontics, Universidad Alfonso X El Sabio, Madrid, Spain; 2 Madrid Health Service, Madrid, Spain; 3 Centro Odontológico de Innovación y Especialidades Avanzadas, Calle de Albarracín, 35, 28037 Madrid, Spain

**Keywords:** Orthodontics, Dentistry, COVID-19, SARS-CoV-2, Bioaerosols

## Abstract

**Background:**

This study evaluates the particle dispersion involved in dental procedures carried out during orthodontic treatments. Variants such as temperature and relative humidity in the dental cabinet were considered.

**Methods:**

Using a particle counter, a pilot study was conducted, in which 98 consecutive recordings were made during appointments of patients undergoing orthodontic treatments. Temperature, relative humidity and particles present at the beginning (AR) and during the appointment (BR) were recorded. A control record (CR) of temperature, relative humidity and particles present was made before the start of the clinical activity. In addition to conventional statistics, differential descriptive procedures were used to analyse results, and the influence of relative humidity on particle concentration was analysed by statistical modelling with regression equations.

**Results:**

The number of particles present, regardless of their size, was much higher in AR than in CR (*p* < .001). The same was true for relative humidity and ambient temperature. The relationship between relative humidity and particle number was determined to be exponential.

**Limitations of the study:**

The limitations are associated with sample size, environmental conditions of the room and lack of discrimination among the procedures performed.

**Conclusions:**

This pilot study shows that from the moment a patient enters a dental office, a large number of additional particles are generated. During treatment, the number of particles of 0.3 microns—which have a high capacity to penetrate the respiratory tract-increases. Moreover, a relationship between relative humidity and particle formation is observed. Further studies are needed.

## Background

In recent months, the infectious disease COVID-19, caused by transmission of the SARS-CoV-2 coronavirus, has caused a global pandemic with serious consequences at all levels [1, 2]. This disease is known to cause acute respiratory syndrome and pose a public health challenge [3, 4].

One year into the pandemic, there is still some controversy about how the virus uses the airborne route for person-to-person transmission. It has been known since the late nineteenth century that respiratory diseases are transmitted by direct or indirect contact with infectious material, respiratory droplets (Flügge droplets larger than 5 microns) or aerosols [5, 6]. The larger respiratory droplets can spread up to a maximum of about 2 m from the source [5].

However, a person's respiratory emissions are a mixture of mucosalivary droplets and a dynamic multiphase gas cloud. Thus, the larger droplets directly or indirectly contaminate the environment closest to the individual, while the smaller droplets (Wells droplets) evaporate and become aerosols, which can remain suspended in the air, be inhaled and penetrate the lung alveoli because of their small size and mass. The trajectory of this cloud depends on the ambient humidity, temperature, velocity and force of expulsion. These aerosols are currently being held responsible for high pollution rates, mainly in enclosed or poorly ventilated places [5–7].

Lindsley et al. [8] measured the presence of influenza RNA virus in particles coughed up by infected individuals and found that the virus was found in 35% of particles smaller than 4 microns, 23% of particles between 1 and 4 microns and 42% of particles smaller than 1 micron.

Recent studies indicate a greater stability and infectious capacity of aerosols disseminated by SARS-CoV-2–infected individuals compared to those dispersed by SARS-CoV-1.3–infected ones. Van Doremalen et al. [9] obtained similar results regarding the stability of both viruses, thus pointing to the possible transmission of SARS-CoV-2 through aerosols suspended in the air for hours.

Once inhaled, the reach of these particles in the respiratory tree depends on many factors such as lung anatomy, respiratory pattern, presence of respiratory disease and particle size. Smaller bioaerosols can reach deeper into the respiratory tract and be retained longer [10].

By knowing the routes of transmission, appropriate preventive measures designed to minimise transmission of infection can be adopted, bearing in mind that to date, results point to a possible risk of aerosol transmission of SARS-CoV-2 [5, 6].

Conventional prevention measures are aimed at providing protection against respiratory droplets of 5 microns or larger. These include measures based on the use of barriers such as masks, frequent handwashing, maintaining social distance and staying indoors as much as possible. Recently, however, the scientific community has begun to warn of the possibility of aerosol-borne transmission of viruses that cause many respiratory diseases, including SARS-CoV-2 [5, 6].

Dental practices are potentially at risk of transmission for COVID-19 and some protocols have been established in order to make the risk as low as possible. [11–13].

In 2017, 50.3% of the Spanish population acknowledged having visited the dentist on one or more occasions less than a year ago [14]. At dental clinic, it is not possible for patients to maintain constant use of the mask, and the professional works at distances that are within reach of respiratory droplets and aerosols. Considering the numerous visits that occur, some dental procedures have come under scrutiny as procedures that generate large amounts of particulate matter, including small particles [5, 10]. These procedures include those that require the use of turbines, use of ultrasound or debonding of fixed orthodontic appliances [10, 15].

There prevails a tendency to think that orthodontists have a lower risk of disease compared to professionals in other fields of dentistry. However, previous studies have shown that 5 min after the start of fixed appliance removal, there is a large increase in bioaerosol levels as compared to that during pre-procedure baseline levels [10] Therefore, it is not surprising that during this pandemic, telemedicine has been incorporated into dental care protocols. [16].

Due to the lack of knowledge generated by the COVID-19 pandemic, this study aims to assess the dispersion during orthodontic treatments objectively and quantitatively and to facilitate the implementation of evidence-based protocols to treat patients more safely and protect the practitioner.

This study objectively establishes the risk level of inhalating bioaerosols in orthodontic practice. Along with future research, it will help to establish which measures should be implemented during patient care in the dental office. For instance: establishing protocols for air renewal, capacity limitations, the use of air purifying filters and dehumidifiers, clinical performance protocols that pay special attention to those procedures that generate greater particle dispersion etc.

## Methods

A prospective experimental study was carried out in which, using a particle counter, a convenience sample of 98 consecutive shots was collected from patients who attended the clinic of the University Master's Degree in Orthodontics of University Alfonso X EL Sabio, between the 10th of September 2020 and the 4th of December 2020. The study was carried out in a single box located in the centre of the university polyclinic. This site was characterised by a semi-open air-conditioned room on the third floor of the dental specialties building with air conditioning provided by forced air renewal without mixing. The air-conditioning system did not have a split system. The floor was also used for activities related to the master's degree in paediatric dentistry.

This pilot study included all patients with appointments who gave their consent for the study to be carried out. This research followed a protocol in accordance with the Helsinki Declaration for medical research and was approved by the Research Ethics Committee of the Faculty of Health Science of the University Alfonso X El Sabio (approval no.2020-44/006).

The registered procedures are those that are carried out in a fixed appliance orthodontic revision. These include changing arch wires, bonding of fallen brackets, placing accessory attachments for biomechanics, placing ligatures and taking impressions. In all cases, the water–air syringe was used at some point; in some cases, a low-speed rotary instrument without aerosol was used.

In the control group, each day of the experiment, the number of particles contained in 1 L of air was recorded before starting the clinical care activity.

In the experimental group, particle number recordings were made on patients seen consecutively on the days of the investigation.

The patients were treated according to the recommendations issued by the Spanish General Dental Council for dental treatment in times of pandemic [17].

Patients were treated by a single operator to avoid bias due to different uncalibrated operators, and an airborne particle measurement was performed at a height of 1.5 m above the ground and 0.5 m from the patient's head.

Two measurements were made for each patient treated. Prior to any procedure, an initial measurement was made with the patient seated without a mask and without aerosol-generating instruments in operation. The other measurement consisted of a recording of the particles generated every minute during the treatment, choosing the minute in which the maximum particle size of 0.3 micron was present.

In each of the measurements, the particles of 0.3 micron, 0.5 micron, 1 micron, 2.5 microns, 5 microns and 10 microns, temperature (Tª) and relative humidity (RH) contained in 1 L of air every minute were recorded. The device used was the TROTEC® cleanroom particle counter model 220 (ISO 21501-4) calibrated by the manufacturer and adjusted to measure the particles contained in 1 L of air.

To verify the record obtained, the procedures were recorded on video with a count superimposed on the image (recording function of the instrument).

Statistical analysis was performed using the IBM-SPSS-25 software application (IBM Corp; released 2017; IBM SPSS Statistics v 25.0 for Windows; Armonk, NY, USA). Data analysis was performed by using the software program SPSS for Windows (version 25.00; SPSS, Armonk, NY).

## Results

### Descriptive analysis

First, an exploratory and descriptive study of 8 quantitative variables was carried out at 3 different times: (a) control measurement: n = 74, (b) measurement of patients at the beginning of treatment: n = 98 and (c) measurement of the same patients during treatment: n = 98.

The variables were tested for normal statistical distribution using skewness and Kurtosis indices as well as the Kolmogorov–Smirnov (KS) goodness-of-fit test. Additionally, a normal Q-Q plot was used as a visual indicator of fit.

The results of this exploration are summarized in Table 1. The following observations were made:

- In the control measurement, almost all variables collected on the number of particles of different sizes are normally distributed (*p* > 0.05 in the KS test). Likewise, the relative humidity tends towards normality. Only two variables—particles > 10µ and ambient temperature—have significant deviations (*p* < 0.01) although their Q-Q plots reveal that they are quite small in distance to the normal model.
- During the initial measurement of the patient group, some asymmetries were seen in the particle size; nevertheless, the deviations according to the KS goodness-of-fit test, although significant (*p* < 0.05), are slight and therefore tolerable. Only for the 2.5-micron particle size variable, the deviation is highly significant (*p* < 0.01), and the Q-Q graph confirms this lack of statistical normality.
- In the measurements taken during orthodontic treatment, in most of the variables, deviations do not reach statistical significance (*p* > 0.05) in the KS test, which allows us to accept their normality. Only for relative humidity, a significant deviation (*p* < 0.01), confirmed by its Q-Q graph, prevents us from accepting that it follows the Gaussian normal model.

**Table 1:** Exploratory analysis. Adjustment to statistical normality of the variables

Status	Variable	Exploration: Form		KS Test	
		Asimetry	Curtosis	Value	*p*-value
CR	Particles 0.3µ	1.00	2.26	0.09 ^NS^	.515
(N = 74)	Particles 0.5µ	0.49	0.49	0.06 ^NS^	.927
	Particles 1µ	0.64	0.18	0.08 ^NS^	.668
	Particles 2.5µ	0.72	1.92	0.09 ^NS^	.524
	Particles 5µ	0.27	− 0.08	0.11 ^NS^	.288
	Particles 10µ	0.65	0.47	0.22**	.002
	Temperature (ºC)	− 0.35	− 1.30	0.27**	.000
	Relative humidity (%)	0.67	− 0.76	0.17 *	.025
AR	Particles 0.3µ	0.45	− 0.43	0.08 ^NS^	.549
(N = 98)	Particles 0.5µ	1.20	1.05	0.12 ^NS^	.101
	Particles 1µ	1.29	1.20	0.16 *	.010
	Particles 2.5µ	1.28	0.91	0.18**	.003
	Particles 5µ	1.42	2.55	0.14 *	.042
	Particles 10µ	1.95	5.19	0.16 *	.013
	Temperature (ºC)	− 0.09	− 0.16	0.15 *	.019
	Relative humidity (%)	− 0.84	− 0.18	0.20 ^NS^	.001
BR	Particles 0.3µ	0.32	− 0.52	0.07 ^NS^	.696
(N = 98)	Particles 0.5µ	0.75	0.21	0.10 ^NS^	.274
	Particles 1µ	0.78	− 0.09	0.13 ^NS^	.062
	Particles 2.5µ	0.94	0.43	0.13 ^NS^	.083
	Particles 5µ	1.04	0.42	0.14 ^NS^	.052
	Particles 10µ	1.09	0.36	0.15 *	.019
	Temperature (ºC)	− 0.02	− 0.40	0.15 *	.024
	Relative humidity (%)	− 0.81	− 0.09	0.19**	.001

*NS* non-significant deviance (*p* > .05); the variable is normally distributed

*Significant but slight deviation (*p* < .05); the variable tends towards the normal pattern

**Significant severe deviation (*p* < .01); the variable does not conform to normality

Therefore, most of the variables obtained are normally distributed or clearly tend to statistical normality. Owing to this statistical normality, parametric tests were chosen.

Second, we proceeded to carry out a study using classical descriptive statistics for this whole set of variables (see Table 2).

**Table 2:** Descriptive analysis of the variables in each of the 3 measurements

Status	Variable	Centricity			Range (Min./Max.)	Variability	
		Mean	95% CI of the mean	Median		Standard deviation	Interquartile range
CR	Particles 0.3µ	6064.5	5936.1/6265.8	6007.0	4585/91.24	869.0	1049.8
(N = 74)	Particles 0.5µ	1760.1	1690.2/1830.0	1770.0	1192/257.0	301.6	354.5
	Particles 1µ	368.2	347.1/389.3	354.0	210/634	91.1	11.0
	Particles 2.5µ	61.1	56.8/65.3	60.5	18/129	18.4	20.5
	Particles 5µ	5.1	4.6/5.6	5.0	1/12	2.3	4.0
	Particles 10µ	3.2	2.8/3.5	3.0	0/8	1.6	2.0
	Temperature (ºC)	22.2	22.0/22.4	22.0	21.0/23.0 5	0.8	1.0
	Relative humidity (%)	42.8	41.9/43.6	42.0	39.0/52.0	3.6	7.0
AR	Particles 0.3µ	17,558.5	16,015.8/19101.2	16,606.5	4127/39797	7694.8	12,561.3
(N = 98)	Particles 0.5µ	7715.2	6894.6/85.35.8	6571.5	1798/20353	4092.9	5220.0
	Particles 1µ	2077.7	1843.2/2312.1	1728.0	559/5660	1169.6	1448.8
	Particles 2.5µ	471.0	414.2/527.8	377.0	104/1250	283.4	293.5
	Particles 5µ	40.9	35.9/46.0	33.5	7/129	25.2	30.3
	Particles 10µ	27.2	23.7/30.8	24.0	6/96	17.6	21.3
	Temperature (ºC)	22.7	22.5/22.9	23.0	20.0/24.9	1.6	1.3
	Relative humidity (%)	46.3	44.7/47.9	48.2	28.0/58.8	8.0	10.0
BR	Particles 0.3µ	22,726.2	20,840.8/24611.6	21,933.5	4991/45021	9404.1	12,556.5
(N = 98)	Particles 0.5µ	9107.9	8250.0/9965.8	8881.2	2134/22112	4279.1	5690.3
	Particles 1µ	2448.9	22,004.5/2693.3	2379.5	731/5855	1219.0	1730.3
	Particles 2.5µ	566.2	504.0/628.3	546.0	129/1508	309.8	433.8
	Particles 5µ	59.2	52.1/66.4	56.9	10/164	35.6	42.0
	Particles 10µ	37.9	33.5/42.4	36.4	10/95	22.4	27.5
	Temperature (ºC)	23.1	22.9/23.3	23.1	20.0/25.5	1.1	2.0
	Relative humidity (%)	45.3	43.7/46.9	45.6	27.0/60.0	7.9	8.4

### Differential analysis

Owing to the tendency of most of the variables towards normality, parametric tests—1-factor ANOVA—were used, except in the assumptions of non-normality, where the results provided by the parametric tests were checked against their non-parametric alternative—the Mann–Whitey or Wilcoxon test. Statistical significance was present in all the results.

Table 3 shows the comparison of the variables studied at the control time with those studied at the "start of treatment" time. The main findings were that the number of particles, of any size, is much higher in the initial situation than in the control group (*p* < 0.001). There were also significant differences (*p* < 0.01) in both the ambient temperature and relative humidity, which increased with respect to the control measurement.

**Table 3:** Inferential analysis. Intergroup comparison of the variables recorded. During vs. control

Variables	Mean (SD)/Median		ANOVA		Effect size: R^2^	Mann–Whitney test	
	AR (N = 98)	Control (N = 74)	F-value	*p*		Value	*p*
Particles 0.3µ	17,558.5 (7694.8)/16606.5	6064.5 (869.0)/6007.0	163.31**	.000	.490	10.63**	.000
Particles 0.5µ	7715.2 (4092.9)/6571.5	1760.1 (301.6)/1770.0	155.80**	.000	.478	11.11**	.000
Particles 1µ	2077.7 (1169.6)/1728.0	368.2 (91.1)/354.0	157.14**	.000	.480	11.20**	.000
Particles 2.5µ	471.0 (283.4)/377.0	61.1 (18.4)/60.5	154.15**	.000	.476	11.21**	.000
Particles 5µ	40.9 (25.2)/33.5	5.1 (2.3)/5.0	148.21**	.000	.468	11.16**	.000
Particles 10µ	27.2 (17.6)/24.0	3.2 (1.6)/3.0	137.32**	.000	.447	11.23**	.000
Temperature (ºC)	22.7 (1.0)/23.0	22.2 (0.8)/22.0	13.88**	.000	.075	3.61**	.000
Rel. humidity. (%)	46.3 (8.0)/48.2	42.8 (3.6)/42.0	12.10**	.001	.066	4.66**	.000

**Highly significant

It has been found (Table 4) that there are highly significant differences (*p* < 0.001) in the number of particles, of any of the sizes, which are equivalent to effect size again very large and higher than the previous ones (50.0%-57.6%). Since the average values are clearly higher in the end-of-treatment situation, it can be stated that there is a very strong statistical evidence to affirm that at the end of the treatment the number of particles of any size is much higher with respect to the control measurement. At the same time, it is observed that there is also an increase in temperature (*p* < 0.001; large effect: 17.5%), as well as a significant (*p* < 0.05 effect3,7%) but smaller increase in RH.

**Table 4:** Inferential analysis. Intergroup comparison of the variables recorded. During vs. control

Variables	Mean (SD)/Median		ANOVA		Effect size: R^2^	Mann–Whitney test	
	BR (N = 98)	Control (N = 74)	F-value	*p*		Value	*p*
Particles 0.3µ	22,726.2 (9404.1)/21933.5	6064.5 (869.0)/6007.0	230.48**	.000	.576	10.93**	.000
Particles 0.5µ	9107.9 (4279.1)/8195.0	1760.1 (301.6)/1770.0	217.08**	.000	.561	11.20**	.000
Particles 1µ	2448.9 (1219.0)/2091.0	368.2 (91.1)/354.0	214.39**	.000	.558	11.21**	.000
Particles 2.5µ	566.2 (309.8)/474.0	61.1 (18.4)/60.5	195.84**	.000	.535	11.21**	.000
Particles 5µ	59.2 (35.6)/48.5	5.1 (2.3)/5.0	169.99**	.000	.500	11.21**	.000
Particles 10µ	37.9 (22.4)/30.0	3.2 (1.6)/3.0	178.03**	.000	.512	11.25*	.000
Temperature (ºC)	23.1 (1.1)/23.0	22.2 (0.8)/22.0	36.11**	.000	.175	5.42**	.000
Rel. Humidity. (%)	45.3 (7.9)/47.5	42.8 (3.6)/42.0	6.46*	.012	.037	4.04**	.000

*Significant

**Highly significant

Finally, when comparing the average values of the measurements taken at the end of the session with those at the beginning (Table 5), highly significant differences (*p* < 0.001) are still found in all the variables, with the values always being higher at the end; except in HR where they are equal or lower. The effect size expressing the magnitude of the changes is much higher in the smallest particles variable (56.8%) compared to the effects of the rest of the variables (< 30%) although always being large or very large effects (between 17.7% and 29.1%). Consequently, we have very solid statistical evidence to be able to affirm that the number of particles increases at the end of the session with respect to the beginning; especially of the smallest ones (< 0.3µ). Likewise, we have sufficient evidence to affirm that the temperature increases (*p* < 0.001; effect of 28.8%) and that the RH. is reduced (*p* < 0.001 and effect of 26.7%).

**Table 5:** Inferential analysis. Intra-group comparison of variables recorded. During vs. initial

Variables	Mean (SD)/median		ANOVA		Effect size: R^2^	Mann–Whitney test	
	BR (N = 98)	AR (N = 98)	F value	*p*		Value	*p*
Particles 0.3µ	22,726.2 9404.1)/21933.5	17,558.5 (7694.8)/16606.5	127.33**	.000	.568	8.52**	.000
Particles 0.5µ	9107.9 (4279.1)/8195.0	7715.2 (4092.9)/6571.5	33.47**	.000	.257	5.99**	.000
Particles 1µ	2448.9 1219.0)/2091.0	2077.7 (1169.6)/1728.0	26.88**	.000	.217	4.93**	.000
Particles 2.5µ	566.2 (309.8)/474.0	471.0 (283.4)/377.0	20.92**	.000	.177	4.34**	.000
Particles 5µ	59.2 (35.6)/48.5	40.9 (25.2)/33.5	39.89**	.000	.291	5.76**	.000
Particles 10µ	37.9 (22.4)/30.0	27.2 (17.6)/24.0	35.46**	.000	.268	5.86**	.000
Temperature (ºC)	23.1 (1.1)/23.0	22.7 (1.0)/23.0	39.19**	.000	.288	5.38**	.000
Rel. humidity. (%)	45.3 (7.9)/47.5	46.3 (8.0)/48.2	35.36**	.000	.267	5.37**	.000

**Highly significant

If we compare the time of treatment with the control (Table 6), we find highly significant differences (*p* < 0.001) in the number of particles, of any size, that are equivalent to the effect size, again very large and higher than the previous ones. Therefore, we can state that during the treatment, the number of particles of any size is much higher than that during the control measurement. Temperature increased greatly from the time of control to the time of treatment and relative humidity increased at a lower rate than that existed before.

**Table 6:** Correlational analysis. Potential Regression Models, predictors of the number of particles of each size, from the relative humidity. Initial situation. (N = 98)

Predictive variable	R^2^ adjust	Contrast test		Coefficient	SE
		Value	*p*-value		
Particles 0.3µ	.998	219.03**	.0000	2.53	± 0.427
Particles 0.5µ	.998	207.30**	.0000	2.31	± 0.432
Particles 1µ	.996	164.21**	.0000	1.96	± 0.452
Particles 2.5µ	.994	125.44**	.0000	1.57	± 0.474
Particles 5µ	.975	61.69**	.0000	0.93	± 0.568
Particles 10µ	.972	57.95**	.0000	0.82	± 0.537

**Highly significant

**Fig. 1:**
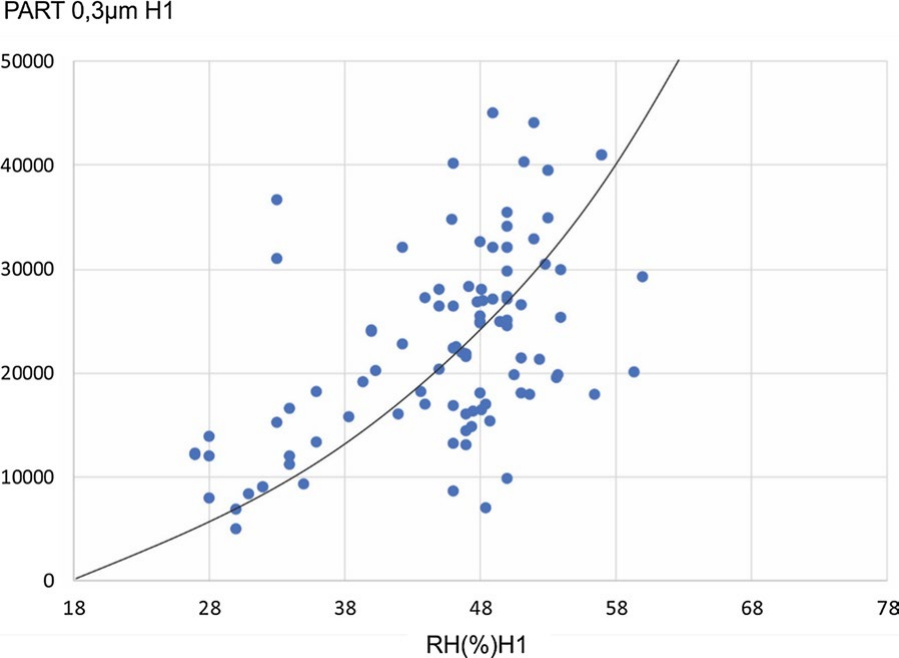
Relationship between 0.3 micron particle size and relative humidity in AR

On comparing the baseline group and the treatment group, we found that particles of all sizes increase significantly while relative humidity remains the same or decreases and the temperature increases. The variations among the smallest particles (0.3 micron) are especially significant (Fig. 1).

To test the correlation between relative humidity and particle size, statistical modelling with General Model Regression Equations was used. The degree of fit of the data to predictive models was checked with the following relationships: linear, quadratic, cubic, logarithmic, inverse, potential and exponential.

Table 6 contains the results of the prediction of the number of particles of different sizes measured in the AR situation from the current relative humidity value in the room. These results, first of all, constitute a significantly strong statistical proof of the direct relationship between relative humidity and the number of particles, i.e., the higher the relative humidity in a room, the more the number of particles. This relationship is not linear but potentially of the type Y = Xb ± ε. In the models presented, the degree of adjustment of this type of mathematical model to the empirical data collected in the initial measurement situation appears significant (always above 97% and even above 99%). From particle size of 5 microns onwards, the smaller the particle size, the better the fit, with best fit at the smallest particle size of 0.3 and 0.5 micron. The potential coefficients (b) of these models decrease in value as the particle size increases, while the margin of error increases.

The results of the same study carried out with the variables collected from BR are presented in Table 7. As can be seen, despite the significant variations between the two measurement situations, the results are comparable. In other words, a potentially direct relationship between the relative humidity of the room and the number of particles observed is maintained. Both values of the coefficients and the standard errors of each of the models are comparable to those obtained in AR.

**Table 7:** Correlational analysis. Potential Regression Models, predictors of the number of particles of each size, from the relative humidity. Situation during. (N = 98)

Predictive variable	R^2^ adjust	Contrast test		Coefficient	SE
		Value	*p*-value		
Particles 0.3µ	.998	227.11**	.0000	2.61	± 0.433
Particles 0.5µ	.998	197.19**	.0000	2.37	± 0.452
Particles 1µ	.997	167.64**	.0000	2.02	± 0.454
Particles 2.5µ	.994	122.67**	.0000	1.63	± 0.500
Particles 5µ	.980	68.74**	.0000	1.03	± 0.563
Particles 10µ	.977	63.74**	.0000	0.92	± 0.540

**Highly significant

Having shown that there is an increase in the number of particles during the treatment with respect to the number that existed during initial measurement, we proceeded to determine the relationship between relative humidity and this trend. To this end, a variable was generated that expresses the magnitude of the difference in the values of the change in the number of particles of each size. Subsequently, the relationship of these differential variables with relative humidity, both in AR and BR, was studied. The best fitting model is the linear model, with direct correlation, i.e., a higher value of relative humidity corresponds to a greater differential increase (BR-AR) between the number of particles. The fits of these models are significantly lower than those found in the previous models but still maintain their high significance, especially for smaller particle sizes.

## Discussion

In the present study, the concentration of aerosols was measured during routine procedures in an orthodontic appointment, bearing in mind that the risk of virus transmission depends on the number of particles and their properties.

From the results obtained in this study, the number of particles, especially the smaller ones, increases from the time of control with an empty room compared to the time of the beginning of the treatment. At this point, no aerosol-generating device had yet been used, suggesting that the changes in particle number were related to unintentional particle input by the operator and/or the patient. This variation of more than 300% in the number of particles is important as it indicates that some of the measured particles are not exclusively generated by the treatment. Whether or not they contribute to an infectious environment is more difficult to determine. Some of the particles found at the initial stage may be the product of respiratory acts and therefore may also be infectious [18]. In such acts, temperature and relative humidity play a modulating role in the formation and number of particles of different sizes.

During the procedures performed, in which air and water syringe and slow handpiece without irrigation were used, which are considered procedures capable of generating aerosols on their own, the particles increased equally. However, these particles have a different characteristic from those of the previous group, and they may have been in contact with the patient's own infectious particles located in the saliva and respiratory tract. In dentistry, the importance of aerosol control for the prevention of cross-infection of health care personnel has been known for many years [19]. Most articles refer to the characterisation of aerosol in restorative procedures and dental surgery. One of the main limitations when investigating dental aerosol is that almost all the articles have characterised it as an element that transmits bacteria, rather than studying it, possibly due to technical difficulties, as an element that transmits viral pathogens [20, 21]. The possible spread of bacteria such as *Mycobacterium tuberculosis* would therefore only imply the need for the widespread use of FFP2 masks instead of the commonly used surgical masks [21].

Within the field of dentistry, the COVID-19 era and its transmission routes have reignited the debate on the argument that the orthodontic treatments pose the least risk to the practitioner [10]. Thus, although general dentists, especially oral and maxillofacial surgeons, were reported as high-risk practitioners at the beginning of the pandemic by the US Department of Labor, they did not fall into the high-risk head group [11, 22].

It is worth noting the orthodontists had to continue caring for patients who had previously started treatments that could not be postponed, as can be done for other dental treatments, and required supervision and maintenance [12]. This itself increased the risk of infection for orthodontists during a period of ignorance about the routes of transmission of the virus and a shortage of appropriate personal protective equipment. Studies should be carried out to assess the risk for each dental specialty [23].

In this context, an important finding has been that with increasing temperature, the size of the generated particles decreases, probably due to dehydration of the larger particles. Size is an important factor affecting the possible transmission of SARS-CoV-2. However, despite all the progress that has been made in understanding the routes of transmission of SARS-CoV-2, airborne transmission is not fully understood and recognised. Since the size of the virus is between 60 and 140 nm, the virus has potential presence and permanence in aerosols [24, 25].

Significantly high SARS-CoV-2 viral loads have been found in the saliva of coronavirus-positive patients, even in asymptomatic patients [26]. Currently, little importance is given to smaller particles, not since these can remain in suspension for long periods of time compared to the 30 s that particles larger than 10 microns are retained [21]. Thus, the importance of direct transmission caused by larger particles at proximity to the source of contamination or transmission via fomites is acknowledged, but less direct and later transmission caused by smaller particles is underestimated [6, 13, 27].

Given the debate on what effect different seasons would have on the development of the pandemic, with the characteristic changes in temperature and humidity, this study was conducted from September to December. This resulted in variations in outdoor and indoor relative humidity that should be correlated to determine the influence of climate on particle dispersion [2, 18, 28].

The influence of relative humidity and temperature on virus survival in aerosols is a controversial issue. For more than 50 years, it has been advocated that increasing indoor relative humidity decreases virus survival [18]. In contrast, Dbouk and Drikakis [29] used 3D computer simulations to indicate that viruses survive in environments with high relative humidity. Thus, a combination of high temperature and low relative humidity leads to a significant reduction in virus viability and penetration.

The effects of relative humidity and temperature should be investigated to limit disease transmission and to avoid future surges caused by exposure to bioaerosols [30, 31]. The role of social distance, the use of appropriate masks and the control of indoor air quality are essential factors to reduce virus transmission in both public and private places [4, 30–32].

In the context of the sudden onset of the pandemic, there is still little literature on the viability of SARS-CoV-2 in aerosols, but it has been estimated that it is possible to maintain infectivity for hours indoor (9, 26, 32).

## Conclusions

This pilot study shows the significant increase in number of particles starting from the moment the patient enters the dental cabinet and removes the mask compared to those that existed during the basal environmental particles that may be found earlier. Regarding the particles generated during treatment, the main finding is an increase in particles of 0.3 micron.

The number of particles, especially smaller ones, increases from the time of control time with an empty room compared to the beginning of the treatment time (300%). This is important as it indicates that some of the measured particles are not exclusively generated by the treatment.

In addition, a correlation between the relative humidity of the medium and particle formation is observed, with greater formation of particles of different sizes when conditions are more humid, which is reported to be more favourable for virus survival.

One of the possible measures to be considered is the modification of environmental settings through climate control. Increased temperature can lead to dehydration and a decrease in the size of the particles generated compared to other large particles. In addition, these particles modelled in some studies may have been inactivated earlier under conditions of increased temperature due to dehydration as well as possibly due to pH changes.

Further studies are needed to establish which measures should be implemented during patient care in the dental office, such as protocols for air renewal, capacity limitations, use of air purifying filters, use of dehumidifiers, conducting the study in clinics with different ventilation systems or in different geographic locations with different climatic condition.

## Data Availability

The data that support the findings of this study are available from the corresponding main author, upon reasonable request.

## Abbreviations

AR: At the beginning
BR: During the appointment
CR: Control record
RH: Relative humidity
